# The NIH is a sound investment for the US taxpayer

**DOI:** 10.7554/eLife.106710

**Published:** 2025-03-25

**Authors:** Nicholas W Gilpin

**Affiliations:** 1 Department of Physiology, Louisiana State University Health Sciences Center New Orleans United States

**Keywords:** Science Under Threat in the United States, science, politics, science funding, National Institutes of Health, basic research, indirect costs, None

## Abstract

Research funded by the National Institutes of Health is essential for improving the health of Americans and developing new drugs and treatments for a wide range of diseases.

The annual budget of the National Institutes of Health (NIH) – the biggest funder of biomedical research in the world – is determined by the US Congress, and in fiscal year 2024 the NIH had a budget of $48 billion. Although $48 billion is a very large sum of money, it is a drop in the ocean compared with the $4500 billion that is spent on health care in the US every year. In the war against disease and suffering, the NIH is vastly underfunded.

Recently, NIH-funded research has come under a new level of fiscal scrutiny, with the newly established Department of Government Efficiency (DOGE) proposing major reductions in specific categories of NIH research funding. The proposed cuts, which are currently mired in litigation, have sent shock waves through the biomedical research world, and have led to hiring freezes, job cuts and uncertainty about the future of biomedical research in the United States.

At the core of these cost-cutting measures is one fundamental question: Is NIH-funded research a worthwhile investment for the US taxpayer?

The answer is yes.

## Improving health and tackling disease

Research funded by the NIH improves health and mitigates disease. For example, NIH-funded discoveries on the link between tobacco use and lung cancer led to public policy interventions and health practices that resulted in sharp drops in lung cancer rates in the US over the last 50 years. NIH-funded research has also contributed to the discovery, development and optimization of many of the most used and top-selling drugs worldwide. These include but are not limited to insulin, anti-cancer drugs, GLP-1 agonists, blood thinners, anti-viral medications, anti-inflammatory drugs and smoking cessation therapies. Due to advances in antiretroviral therapies, for example, people diagnosed with HIV can now expect to live almost as long as the average US citizen.

A major ongoing challenge is that the population is rapidly aging, and many of the conditions that cause the most death and disability in the US – heart disease, cancer, addiction and mental health diagnoses, diabetes, low back pain, pulmonary disease, stroke, kidney disease and dementia – are more prevalent in aged individuals. With the number of Americans over the age of 65 projected to increase from 58 million in 2020 to 82 million in 2050, one could argue that NIH-funded research is more important now than it has ever been.

## NIH is essential for the biomedical research enterprise

When one considers the vastness of biomedical research, spanning everything from cardiovascular disease to pancreatic cancer to dementia to chronic pain, it becomes clear that no single campus (for example, the NIH campus in Bethesda) can perform all of this work. Many labs with specialized and diverse expertise are required, so performing these research projects at universities across the country has long been the agreed-upon solution.

It has been suggested that the private sector can replace the work that NIH does, but because the private sector is concerned mainly with profits, this simply would not work. Look no further than the abandonment of neuroscience R&D by several major pharmaceutical companies due to high drug development failure rates. This gap in research and development is filled by the NIH, with more than half of all basic research occurring at universities or within the federal government. Furthermore, NIH-funded labs train the individuals who go on to become the private sector biotechnology and pharmaceutical workforce. Basic research and scientific training are supported by the NIH, which is financially backed by an investment from the American taxpayer. The basic biomedical research funded by NIH sets the stage, and is indeed essential, for later private sector investment.

Some of the success stories of NIH-funded research have been serendipitous and others have been incremental, emphasizing that risky and sequential science are both extremely important. Furthermore, taxpayer dollars should be used to improve the health of all Americans. For this reason, we use both males and females in our animal work, and mixed demographics in our human work (as mandated by the NIH). It is also critical to address health and disease issues that are present only in some populations (e.g., women’s health), and those which are more prevalent in specific populations (e.g., environmental pollutants in specific areas). Failure to do so leads to treatment gaps and blind spots.

## Driving the economy and creating jobs

Given the opportunity to invest one dollar and receive a guaranteed $2.56 in return, most Americans would likely do so without hesitation. That investment opportunity already exists at the NIH. In an independent study performed by United for Medical Research in 2024, it was reported that every dollar invested in the NIH returned $2.56 in economic activity, and that NIH funding produced $94.58 billion in new economic activity across all 50 states.

Furthermore, NIH funding supported more than 400,000 jobs in fiscal year 2024. These jobs are not only filled by scientists, but also by IT staff, compliance officers, technicians, administrative assistants, finance personnel, maintenance staff and workers performing other roles. Most Americans likely know at least one person who works one of these jobs. Indeed, small American cities and towns with large universities depend on these jobs.

## NIH-funded research is rigorous and competitive

Along with basic science, large portions of NIH funding go to clinical trials, community outreach, health education and epidemiological work. The scientists who perform this work receive extended training, usually 4–6 years for a highly specialized PhD, followed by 4–6 years of postdoctoral training, which is also highly specialized. Likewise, the small number of researchers who secure faculty positions must survive a highly competitive grant submission and award process where failure rates are usually between 80 and 90%.

When researchers at universities and other research institutes apply for NIH grants, their applications are vetted by scientific experts, usually several times, and they are also reviewed by NIH staff to ensure that they fit with NIH priorities and comply with various regulations. About 83% of the NIH budget is spent on this “extramural” research (occurring outside of NIH facilities, usually at universities). The competition for NIH grants is intense and this organically creates a high level of rigor in biomedical research, because non-rigorous work does not receive funding.

After a project is funded, the researchers must comply with federal regulations that protect human subjects, animal subjects and scientists themselves, and must also exercise budgetary oversight and fiscal responsibility. The costs of doing this – along with the other costs, such as heating, lighting, IT and so on – are funded by NIH indirect costs (also known as “overheads”). Indirect costs are calculated as a percentage of the direct cost of a grant, and this percentage is based on the costs of performing this work, and so it varies from one university to another: for example, building leases and salaries in San Francisco are higher than those in Iowa City, so the indirect cost rate is also higher.

On February 7, 2025, the NIH announced a policy that aims to cut indirect cost rates for all new and existing grants. Reducing indirect cost rates to 15% across the board will be catastrophic if the regulations that researchers must comply with remain unchanged. Some have proposed that cuts to indirect costs may be used to fund more scientific projects or otherwise reallocated to direct costs. Ironically, but logically, more awards or higher operational budgets will lead to increases, not decreases, in the activities that are funded by indirect costs. Others have proposed that indirect costs could be built into the direct costs of research projects, but this would lead to instability and chaos because these jobs cannot be done on a “per-project basis.” The people who do these jobs – such as compliance officers, IT staff, and animal care technicians – must be trained and the roles must be continuously staffed over time. Funding the salaries of the people who fill those roles using indirect costs is clearly the more efficient strategy, and efficiency is the goal after all.

## Where do we go from here?

It is clear that scientific communication by scientists, universities, and research societies must drastically improve. Biomedical research and the country at large would benefit from specific forums devoted to the communication of scientific and biomedical discovery to the general public. We need funded initiatives to facilitate this, and we need individuals devoted to doing this work. Those individuals should be compensated for their work, but they should not commercialize that work or compete for advertising dollars. Such conflicts of interest strain credulity, slant the science, and dangerously blur the line between science and sales.

While some reform to funding structures may be in order, indirect cost rates must be sufficient to keep universities in the business of supporting biomedical research. This is good not only for the health of the nation and the economy, but also for national security and America’s position as a global leader in science and technology. Healthy Americans can work, innovate, contribute to their communities and raise families.

Finally, NIH-funded research should reflect health issues in America, improve overall health, and improve disease outcomes. If American citizens are convinced that they benefit from the NIH-funded biomedical research enterprise, they should be vocal and persistent in communicating this to their government representatives. Biomedical research funded by the NIH is critical for the health of the United States and its citizens.

